# Phase transitions in disordered mesoporous solids

**DOI:** 10.1038/s41598-017-07406-2

**Published:** 2017-08-03

**Authors:** Daniel Schneider, Daria Kondrashova, Rustem Valiullin

**Affiliations:** Felix Bloch Institute for Solid State Physics, University of Leipzig, Leipzig, Germany

## Abstract

Fluids confined in mesoporous solids exhibit a wide range of physical behavior including rich phase equilibria. While a notable progress in their understanding has been achieved for fluids in materials with geometrically ordered pore systems, mesoporous solids with complex pore geometries still remain a topic of active research. In this work we study phase transitions occurring in statistically disordered linear chains of pores with different pore sizes. By considering, quite generally, two phase change mechanisms, nucleation and phase growth, occurring simultaneously we obtain the boundary transitions and the scanning curves resulting upon reversing the sign of the evolution of the chemical potential at different points along the main transition branches. The results obtained are found to reproduces the key experimental observations, including the emergence of hysteresis and the scanning behavior. By deriving the serial pore model isotherm we suggest a robust framework for reliable structural analysis of disordered mesoporous solids.

## Introduction

Better understanding of thermodynamics of mesoscopic systems, in particular of molecular systems confined to mesopore spaces, is important for very diverse fields including atmospheric and environmental sciences, food preservation, and nanotechnology. Due to a beneficial combination of high specific surface area and favorable transport properties mesoporous solids are currently considered as attractive host materials for use in practical applications such as catalysis, sensing, and medicine. Hence, a thorough understanding of fluid behavior in mesopore spaces is an important basis for their technological usage and for improvement of structure characterization methods for mesoporous solids. Notably, the most widely used characterization approaches, such as gas sorption and thermoporometry, are based on the measurements of the pore size-dependent alterations of the phase transition points of confined matter^1, 2^. In the recent decades, significant progress in the understanding of the confinement effects was facilitated by the emergence of mesoporous solids with ordered porosities^3–5^. The experimental results obtained with these materials and advancements of theoretical approaches majorly contributed to improve our knowledge about the microscopic processes occurring in porous materials with simple pore structures^2, 6–13^. At the same time, phase transitions in porous solids with complex pore architectures still remain poorly understood.

**Figure 1:**
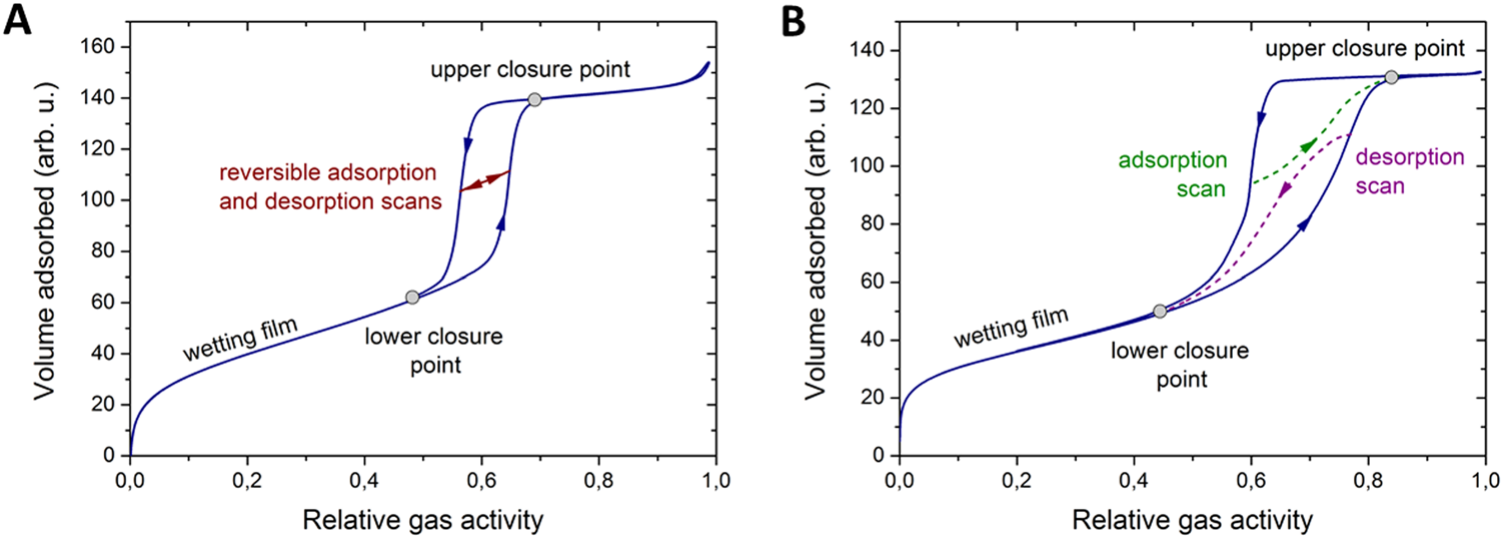
Phase transition patterns in ideal and disordered pores. Schematics of the gas sorption isotherms typically observed in the experiments with mesoporous solids with (**A**) cylindrical pore geometry, such as MCM-41, and with (**B**) disordered pores, such as Vycor porous glass.

It is well recognized that phase transitions in materials with ordered and disordered pore systems differ substantially. As an example, Fig. 1 shows schematically gas sorption isotherms typically obtained at sub-critical temperatures for porous solids with cylindrical pore geometries, like MCM-41 or SBA-15^14^, and with random materials, like Vycor porous glass^15, 16^. Similar patterns are also reported for melting and freezing in confined spaces^17–22^. In both cases shown in Fig. 1A,B there is irreversibility between the capillary-condensation (gas-liquid) and evaporation (liquid-gas) transitions. However, the shapes of the hysteresis loops are found to be substantially different. Thus, in cylindrical pores the two transition branches are parallel to each other and the irreversibility is known to be caused by metastability along the adsorption branch. The two transition branches will also remain to be parallel to each other for a collection of independent channels with different pore diameters, but they will appear not as steep as for one single channel. In contrast, the hysteresis loop in disordered materials is often found to be asymmetric with both transitions being dominated by complex free-energy landscape^23, 24^. Even stronger divergences are found for the scanning curves, i.e. curves obtained by reversing the sign of the evolution of the gas pressure at different points along the main transition branches. In the former case (uniform pores) the adsorption and desorption scans cross between the boundary curves^25^, while in the latter case (disordered pores) the scanning curves merge at either the hysteresis upper or lower closure points. Interestingly and somewhat intriguingly, the patterns expected for disordered solids have also been observed with ordered MCM-41 and SBA-15 materials^26, 27^. Similarly, disordered porous solids may also exhibit the transition patterns resembling that of ideal materials^28^.

The independent domain theory developed by Everett for capillary phenomena^15^, also referred to here as the independent pore model (IPM), assumes that all pores with different pore sizes fill and empty independently from each other. The respective transition pressures for each pore size can be predicted either using classical theories or more advanced density functional theories (DFT) and simulation approaches. Combined with the Barret-Joyner-Halenda (BJH) calculation scheme^29^, IPM still remains the dominant approach for deriving the pore size distributions (PSD) of mesoporous materials irrespective of their pore space organization. Unveiling the pore connectivity effects is not a trivial problem and different strategies tackling this problem have been reported in the literature. Thus, capillary-condensation and evaporation transitions were studied using disordered lattice-gas models and the majority of features seen in the experiments were reproduced^23, 30^. Ink-bottle pore systems of different complexity, representing the elementary units of statistically disordered materials, were investigated using different computer simulation approaches^6, 31, 32^. Long linear chains^33^ and networks^34–36^ of pores with different pore sizes were also addressed in numerical simulations and some important insights into, e.g., behavior of the scanning curves were obtained. Theoretical models of capillary phenomena were mostly concerned with the percolation theories in which the connectivity effects were modeled with the help of the loopless Bethe lattices^14, 37, 38^. This later approximation is typically used for it allows for analytical solutions of the percolation-related problems^39^. It was shown that a qualitatively reasonable behavior of the capillary condensation and evaporation transitions as well as of the scanning behavior can be obtained in this way.

Being useful for the exploration of some microscopic aspects accompanying the phase transitions, all these approaches cannot however be easily implemented for the pore size analysis. At the same time, it has been evident in the literature that already the simplest model representing random porous materials, namely a linear chain of pores with different pore sizes, in what follows referred to as the serial pore model (SPM), exhibits the key features observed in the experiments as exemplified by Fig. 1B ^33, 40, 41^. Similarly to the Cayley trees, linear pore chains in SPM are also loopless. Hence, the percolation problem for SPM can be solved exactly. Moreover, the analysis can also be performed for scenarios in which the percolation-like transitions occurring upon variation of the chemical potential are further complicated by the occurrences of the phase nucleation processes. In the present work we report the rigorous results obtained within this model for phase equilibria in disordered pore systems. The equations derived are generally applicable for predicting different phase equilibria, including gas-liquid and liquid-solid transitions. The latter is validated using computer simulations of the respective models. Moreover, this formalism is similarly applicable for similar phenomena exhibiting pore-size dependent features. These may include liquid-liquid separation and solid-solid transitions in confined space and mercury intrusion being the most representative examples. As an important point, the final analytical results resemble the BJH scheme for the pore size analysis using IPM, but applied to SPM. Therefore, they provide a more accurate route for structural characterization of disordered porous materials.

## Results

### Phase transitions in statistically disordered porous solids

In this work we consider one-dimensional chains of cylindrical channel sections with different pore (channel) diameters *ξ* and with equal length *l*_0_ joined to each other. *ξ* are distributed as determined by a normalized pore size distribution function (PSD) *ϕ*(*ξ*) $({\int }_{0}^{\infty }\varphi (\xi )d\xi =1)$. The total channel length measured in units of *l*_0_ is *L*. The pore diameters of two adjacent pore sections are statistically uncorrelated, i.e. the probability that any arbitrarily selected pore section has a diameter *ξ* is equal to *ϕ*(*ξ*).

The evolution of the phase composition in the pores upon variation of the systems chemical potential *μ* depends on the governing phase transition mechanisms. It is implied here that more than one mechanism may act in parallel. It is considered, in particular, that the phase composition may change (i) by nucleating the new phase in a pore section (in what follows this mechanism is referred to by the subscript ‘n’) and (ii) due to growth (subscript ‘g’) of the already formed domains of the new phase into the adjacent pore sections. In the first scenario (i), the nucleation is accompanied by immediate filling of the entire pore section considered with the phase nucleated. The probability for a transition to occur in a randomly selected pore section is determined by the chemical potential *μ*, pore diameter *ξ*, and phase state in the adjacent pore sections. These probabilities for each transition mechanism can independently be found from the respective kernels, namely theoretically or experimentally obtained curves providing the relationships between the pore size and the chemical potential *μ*_*tr*_ at which the transition occurs^18, 42, 43^. Instead of *μ* it is more convenient to use the thermodynamic quantity varied in the experiment. It can be, e.g., gas pressure or temperature and in what follows will generally be referred to as *χ*.

The main objective of this work was to establish the average phase composition as a function of *χ* and of the history how a particular value of *χ* was attained. For the sake of convenience, we denote the phase pair constitutes by ‘L’ and ‘G’, with ‘L’ denoting the new phase filling the pore space upon increasing *χ*. ‘L’ may be associated with the capillary-condensed phase in gas sorption, with the molten liquid phase in thermoporometry experiments, and with the displacing wetting phase in mercury intrusion. ‘G’ denotes respectively the phase replaced by ‘L’ and may be associated with the gaseous or ice phases. The quantities typically measured in the experiments are the relative volume fractions occupied by one of the phases. In what follows, we evaluate the fraction *θ* of the pore sections containing the phase ‘L’. Hence, *θ* resembles directly the experimentally measured normalized sorption isotherms and melting/freezing and mercury intrusion curves.

### Boundary hysteresis loops

Let us first evaluate *θ* obtained upon a monotonous increase of *χ* starting from a state with all pores containing the phase ‘G’ to a state when all pores will contain ‘L’. Let us introduce the function *f* describing whether at a given *χ* a pore section with the pore size *ξ* can be filled by the phase ‘L’ via nucleation or via phase growth. It is convenient to define *f* as

1 $${f}_{i}(\chi ,\xi )={\rm{Heaviside}}(\chi -{\chi }_{i,tr}(\xi ))$$

where the subscript ‘i = (n, g)’ refers to the transition mechanism and *χ*_*i*,*tr*_ is determined by the corresponding kernel. With this definition *f*_*i*_ = 1 means that, for given *ξ* and *χ*, the phase change can occur, while *f*_*i*_ = 0 implies that the transition is improbable. The mean probabilities *p*_*i*_(*χ*) for the transition ‘G → L’ to occur in an arbitrarily selected pore section via one of the two mechanisms are readily found as^16, 37^

2 $${p}_{i}(\chi )={\int }_{0}^{\infty }{f}_{i}(\chi ,\xi )\varphi (\xi )d\xi ={\int }_{0}^{{\xi }_{cr,i}}\varphi (\xi )d\xi ,$$

where ${\xi }_{cr,i}\equiv {\chi }_{i}^{-1}(\chi )$ is the size of the largest pore in which the transition is allowed at given *χ*. By introducing the cumulative pore size distribution ${\rm{\Phi }}(x)={\int }_{0}^{x}\varphi (\xi )d\xi$, equation 2 takes a simple form

3 $${p}_{i}(\chi )={\rm{\Phi }}({\xi }_{cr,i}\mathrm{)}.$$

The mean number of the pore sections (per channel) in which the phase ‘L’ can be created by nucleation is *p*_*n*_(*χ*)*L*. Hence, the number *n*(*χ*) of seeds of the phase ‘L’ , which may further initiate phase growth, is

4 $$n(\chi )={p}_{n}(\chi )L+{n}_{b}.$$

By introducing *n*_*b*_ in equation 4 we model the thermodynamic conditions at the channel openings. In particular, *n*_*b*_ = 0 and *n*_*b*_ = 1 − *p*_*n*_(*χ*) assure that, at the channel openings, the direct contact with the bulk phases ‘G’ or ‘L’ is provided, respectively. The former condition implies that only nucleation of the new phase can trigger the phase change in the pores, while, in the latter scenario, the phase ‘L’ is already supplied at the channel openings, hence it can invade into the channels. Each pore section in which ‘L’ was nucleated, including two pore openings in the case of *n*_*b*_ = 1 − *p*_*n*_(*χ*), may act as a seed giving rise to the phase growth in the axial direction by sequential filling of the adjacent sections by the phase ‘L’. The growth probability, i.e. the probability to initiate the phase change in a pore section adjacent to the one already containing the phase ‘L’, is determined by *p*_*g*_(*χ*) and is, as a rule, larger than *p*_*n*_(*χ*). The mean length *λ* (in units of the length *l*_0_) of a domain grown axially in an infinitely long channel can be found as (see Supplementary)

5 $$\lambda (\chi )=2{p}_{g}\frac{1-{p}_{g}^{L\mathrm{/2}}}{1-{p}_{g}}.$$

The relative phase composition *θ* is clearly related to  *n*(*χ*)*λ*(*χ*), that is the number of the seeds of the phase ‘L’ , which can be nucleated at given *χ*, times the average length of each domain grown from these seeds. However, upon deriving equation 5 it was implied that each domain in the channel can grow independently, hence these domains overlap. Therefore, in order to find the correct *θ*, one needs to exclude from *n*(*χ*)*λ*(*χ*) the total length of the overlaps. This is a known problem of placing *n* rods of a length *λ* randomly over an interval *L* and finding the total length of the intervals not covered by the rods^44^. With the known solution of this problem, *θ* may be shown to result as (see Supplementary)

6 $${\theta }_{a}(\chi )=1-{(1-\frac{\lambda (\chi )}{L})}^{n(\chi )},$$

where with the subscript ‘a’ (ascending) we have explicitly indicated that *θ* was obtained upon increasing *χ*. For *L* → ∞, equation 6 further simplifies to

7 $${\theta }_{a}=1-\exp \{-\frac{2{p}_{n}{p}_{g}}{1-{p}_{g}}\}.$$

If *χ* is monotonously decreased, the governing equations remain the same. However, all parameters referring to the phase ‘L’ need now be replaced by the ones referring to the complementary phase ‘G’. All these parameters will, in what follows, be indicated by the prime symbol. The mean transition probabilities ${p}_{i}^{^{\prime} }$(*χ*) and the mean number *n*′(*χ*) of the seeds of the phase ‘G’ can be found as

8 $${p}_{i}^{^{\prime} }(\chi )={\int }_{{\xi }_{cr,i}}^{\infty }\varphi (\xi )d\xi =1-{\rm{\Phi }}({\xi }_{cr,i}^{^{\prime} })$$

and

9 $$n^{\prime} (\chi )={p}_{n}^{^{\prime} }(\chi )L+{n}_{b}^{^{\prime} },$$

respectively, with ${\xi }_{cr,i}^{^{\prime} }$ being now the size of the smallest pore in which the phase change may occur as determined by the corresponding kernel. Once again, because of practical relevance we evaluate the fraction *θ*(*χ*) occupied by the phase ‘L’ ,

10 $${\theta }_{d}(\chi )={(1-\frac{\lambda ^{\prime} }{L})}^{n^{\prime} (\chi )},$$

where the subscript ‘d’ (descending) indicates that *θ* was obtained upon decreasing *χ*. *λ*′ is given here by equation 5 with *p*_*g*_ replaced by ${p}_{g}^{^{\prime} }$. For *L* → ∞ equation 10 simplifies to

11 $${\theta }_{d}=\exp \{-\frac{2{p}_{n}^{^{\prime} }{p}_{g}^{^{\prime} }}{1-{p}_{g}^{^{\prime} }}\}.$$

Finally, equations 6 and 10 compose the so-called boundary hysteresis loop, reminiscent of the adsorption/desorption isotherms in gas sorption, freezing/melting curves in thermoporometry, and intrusion/extrusion curves in mercury intrusion. For monolithic materials, i.e. materials with large *L*, the pair of equations 7 and 11 can be used instead. It is worth mentioning that equations 7 and 11 describe quite generally the conditions when nucleation is effective. The relevant examples here are capillary condensation and cavitation-driven evaporation. If the transition have purely percolation-like character, such as evaporation via gas invasion or mercury intrusion, *p*_*n*_ and ${p}_{n}^{^{\prime} }$ in equations 7 and 11 can be shown to be equal to 1/*L*, hence these equations become, respectively,

12 $${\theta }_{a}=1-\exp \{-\frac{2}{L}\frac{{p}_{g}}{1-{p}_{g}}\}$$

and

13 $${\theta }_{d}=\exp \,\{-\frac{2}{L}\frac{{p}_{g}^{^{\prime} }}{1-{p}_{g}^{^{\prime} }}\}.$$

In what follows, we discuss in more detail the predictions of the model with the help of the lattice-based Monte Carlo simulations of phase transitions in pore systems^45, 46^. We use these simulations for two purposes. First of all, the kernels required for SPM can readily be obtained in the simulations and may further be used to discuss the general predictions. Secondly, the phase equilibria can directly be studied in the simulations by using some models of the disordered pore systems and the results obtained in this way can further be used to validate the theoretical results obtained.

**Figure 2:**
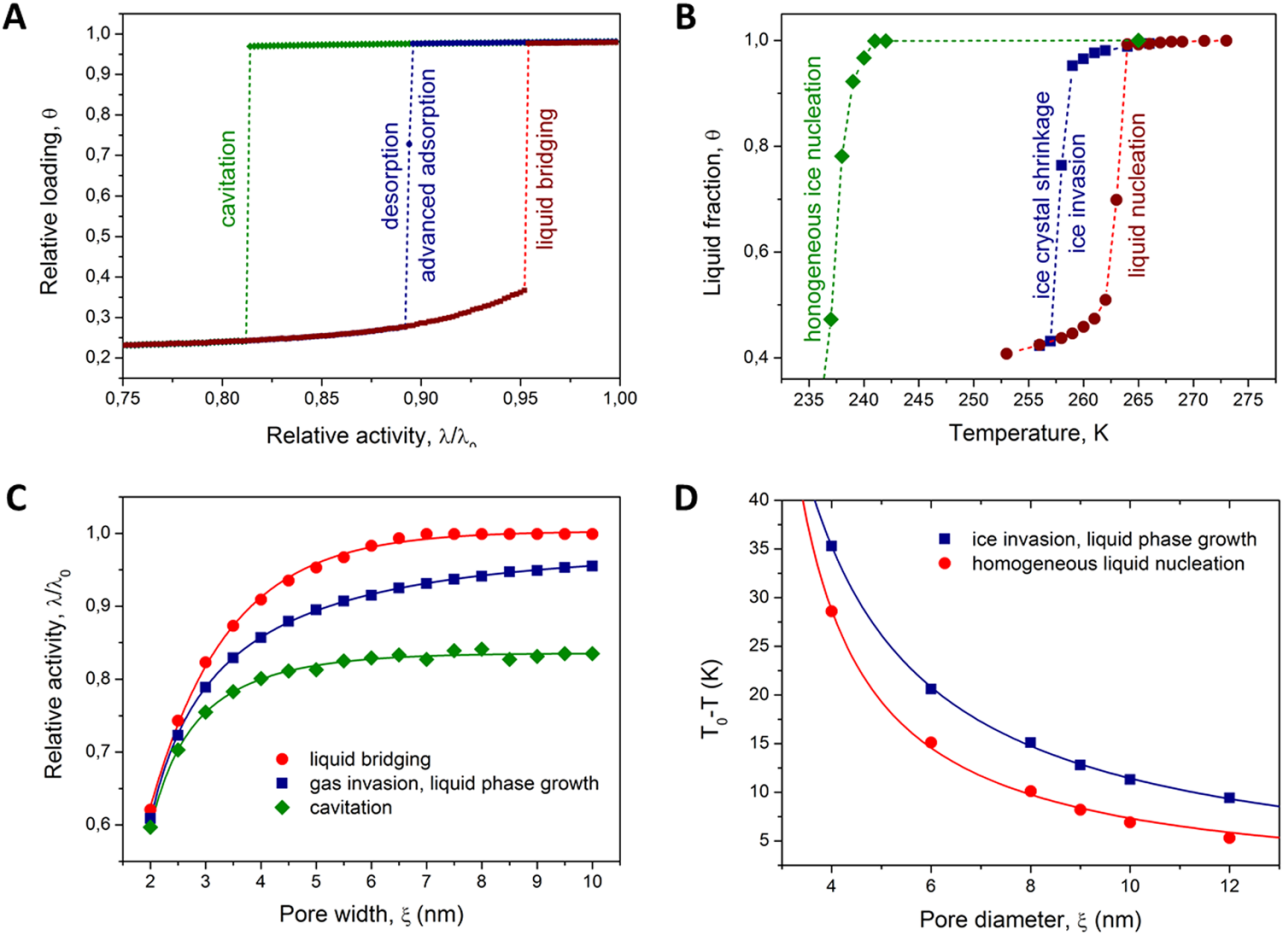
Phase transition in ideal pores and transition kernels. (**A**) Adsorption and desorption isotherms obtained using GCMC simulations of a lattice-gas in a slit pore of 5 nm width as a function of relative activity at *T*/*T*_*c*_ = 2/3. Different transitions are obtained using different pore geometries. A slit pore open at both ends exhibited capillary-condensation (denoted as ‘liquid bridging’) and evaporation (denoted as ‘desorption’) upon increasing and decreasing gas activity, respectively. A capped slit pore exhibited the reversible capillary-filling (‘advanced adsorption’) and evaporation (‘desorption’) transitions. A slit pore with the length of 10 nm and closed at both ends was used to locate the cavitation pressure (‘cavitation’). (**B**) The fraction of the liquid phase in a cylindrical pore with a diameter of 6 nm obtained upon temperature variation for the Kossel crystal. As in (**A**), different pore geometries were used to address different transitions. The channel open at both ends exhibited the irreversible melting (‘liquid nucleation’) and freezing (‘ice invasion’) transitions, while in the capped cylinder two reversible transitions (‘ice crystal shrinkage’ and ‘ice invasion’, respectively) were obtained. The infinitely long cylinder, modeled using periodic boundary conditions, was used to locate the homogeneous nucleation temperature (‘homogeneous ice nucleation’). (**C**,**D**) The gas activities and temperatures at which the different transitions occur in ideal pores (kernels). The solid lines are the best fits of the appropriate analytical expressions used further to compute phase compositions in disordered pores.

To illuminate different aspects of the phase transitions, we perform case studies of gas-liquid and liquid-solid equilibria in disordered pore system. In the former case we considered a lattice-gas confined to slit pores, while in the latter case we model the Kossel crystal in cylindrical channels (see Methods). Figure 2A,B exemplify the typical results obtained with the pores of uniform pore sizes for selected thermodynamic parameters. They show the transition conditions corresponding with the occurrences (i) of the gas or ice invasion from the bulk phase into the pores upon lowering gas pressure or temperature, respectively; (ii) of the growth of the liquid domains upon increasing gas pressure or temperature, respectively; (iii) of the formation of the liquid bridges in the pores containing gas or ice phases upon increasing gas pressure or temperature, respectively; (iv) and of the occurrences of cavitation or homogeneous ice nucleation upon decreasing gas pressure or temperature respectively. Importantly and in complete agreement with the literature reports, the pressures and temperatures at which (i) and (ii) take place are found to coincide^47–49^. The information extracted from the data obtained in this way using different pore sizes were compiled into the respective kernels shown in Fig. 2C,D. In Fig. 2D we did not show the homogeneous ice nucleation temperatures because they were too low and were not attainable in the simulations performed with the disordered pores, i.e. under all conditions studied in the present work freezing in the pores was always exclusively dominated by ice invasion from the pore boundaries.

**Figure 3:**
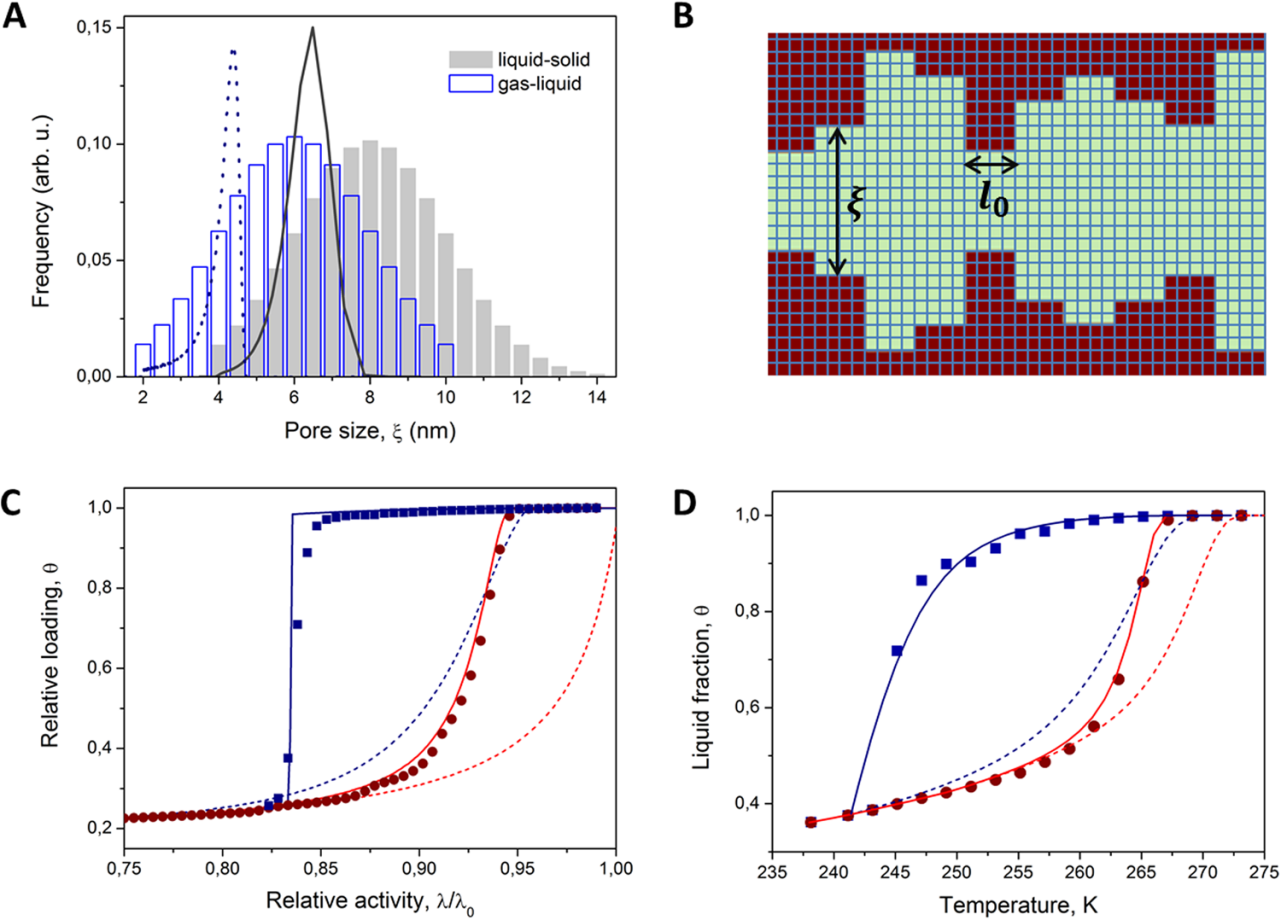
Gas sorption isotherms and freezing and melting transitions in disordered pores. (**A**) The pore size distributions used for the simulations of the gas-liquid (empty bars) and liquid-solid (filled bars) equilibria in disordered pores. The broken and solid lines show respectively the pore size distributions resulting if the adsorption isotherm of (**C**) and melting transition of (**D**), obtained in the simulations, are analyzed using the BJH scheme assuming that all pores are independent from each other (independent pore model). (**B**) Cross-sectional schematic view of a part of a disordered channel used in the simulations. (**C**) Adsorption (circles) and desorption (squares) isotherms obtained using GCMC simulations of a lattice-gas in disordered channels at *T*/*T*_*c*_ = 2/3. (**D**) Melting (circles) and freezing (squares) transitions obtained using Monte-Carlo simulations of the Kossel crystal in disordered pores. In (**C** and **D**) the broken and solid lines show the adsorption and desorption and melting and freezing transitions predicted by the independent and serial pore models, respectively. For the data evaluation the kernels shown in Fig. 2 were used. See Methods for more details on the pore systems studied in the simulations.

The results of the simulations performed with the disordered pores are shown in Fig. 3. In these simulations the long pores were composed of statistically distributed pore sections whose pore sizes were given by PSDs shown in Fig. 3A. Figure 3B shows schematically a part of the typical pore configuration. The relative amounts of the liquid phase obtained for different gas activities (sorption isotherms) and different temperatures (melting and freezing curves) are shown in Fig. 3C,D, respectively. Importantly, the simulation results are found to deviate notably from those predicted by IPM as shown by the broken lines. With the kernels available (Fig. 2C,D), the mean phase compositions obtained upon monotonous changes of the thermodynamic parameters can readily be obtained using Eqs 6 and 10. They are shown in Fig. 3C,D by the solid lines and are found to be in excellent agreement with the simulation data. This agreement proves the robustness of SPM in describing the boundary transitions in disordered pore systems. In turn, the theory would allow a reliable pore size analysis. It may be be noted that for one-dimensional channels with geometric disorder the application of IPM with the BJH analysis scheme yields PSDs significantly underestimating the real pore sizes (see Fig. 3A). The latter finding supports some observations reported in the literature showing that PSDs experimentally obtained using gas sorption or thermoporometry methods for materials possessing geometric disorder delivered pore sizes smaller than those obtained by electron microscopy methods^50, 51^.

### Scanning behavior

In the previous section only the full cycles of the *χ* variation were considered. Let us address now how would *θ* vary if *χ* is varied non-monotonically, i.e. if the variation direction is inverted at some intermediate stages. The family of the thus collected ascending and descending curves are commonly referred to as scanning behavior^15^. It is often argued in the literature that the scanning curves contain more detailed information on both the pore space properties and on the phase transition mechanisms^13^.

Let us first consider an ascending scan started at *χ*_0_ attained upon a monotonous decrease of *χ* to *χ*_0_ along the descending boundary transition line. As the first step, the mean number ${N}_{0}^{^{\prime} }$ of the continuous domains of the phase ‘G’ found in the channels at *χ*_0_ and their mean length ${{\rm{\Lambda }}}_{0}^{^{\prime} }$ need to be obtained. It can be shown (see Supplementary) that, for large systems, these two quantities can be reasonably approximated by

14 $${N}_{0}^{^{\prime} }={n}_{0}^{^{\prime} }{\theta }_{d}({\chi }_{0})$$

and

15 $${{\rm{\Lambda }}}_{0}^{^{\prime} }=(1-{\theta }_{d}({\chi }_{0}))\frac{L}{{N}_{0}^{^{\prime} }}.$$

With this initial configuration, *χ* is now started to increase. The occurrence of the phase transition in these domains of phase ‘G’ can be treated in line with the analysis performed in the preceding section by replacing *L* by ${{\rm{\Lambda }}}_{0}^{^{\prime} }$, and by taking into account that, at the domain boundaries, the contact with the pore sections containing the phase ‘L’ is provided, i.e. with *n*_*b*_ = 1 − *p*_*n*_(*χ*). In addition, because the pore sizes in the sections belonging to these domains represent only a part of the total PSD *ϕ*(*ξ*), the transition probabilities *p*_*i*_(*χ*) need to be redefined. As a reasonable approximation, they can be found as

16 $${p}_{as,i}(\chi )=\frac{1}{Z}{\int }_{{\xi }_{0}}^{\infty }{f}_{i}(\chi ,\xi )\varphi (\xi )d\xi =\frac{1}{Z}({\rm{\Phi }}({\xi }_{cr,i})-{\rm{\Phi }}({\xi }_{0}))$$

where *ξ*_0_ denotes the pore size of the smallest pore not accommodating the phase ‘L’ at *χ*_0_ and $Z={\int }_{{\xi }_{0}}^{\infty }\varphi (\xi )d\xi$ is the normalization constant. Finally, the ascending scanning curves are obtained as

17 $${\theta }_{as}(\chi )={\theta }_{d}({\chi }_{0})+(1-{\theta }_{d}({\chi }_{0}))\theta (\chi ,{\chi }_{0}),$$

where *θ*(*χ*, *χ*_0_) is given by equation 6 with *L* = ${{\rm{\Lambda }}}_{0}^{^{\prime} }$ and other parameters in this equation obtained using *p*_*as*,*i*_(*χ*) as given by equation 16.

The descending scans are obtained in a similar way. Here the initial state at *χ*_0_ is attained upon increasing *χ* along the ascending boundary transition line. One has to find now the mean length Λ_0_ of the continuous domains containing the phase ‘L’ and to model the phase change in these domains by treating them as being separate channels of length Λ_0_ with the phase ‘G’ provided at the domain boundaries. This is once again similar to the already solved problem of the boundary descending curve. The final equation for the descending scanning curves is readily obtained as

18 $${\theta }_{ds}(\chi )={\theta }_{a}({\chi }_{0})\theta (\chi ,{\chi }_{0}),$$

where *θ*(*χ*, *χ*_0_) is given by equation 10 with *L* = Λ_0_. *n*′ and *λ*′ in equation 10 are obtained using the redefined transition probabilities

19 $${p}_{ds,i}^{^{\prime} }(\chi )={\int }_{{\xi }_{cr,i}}^{{\xi }_{0}}\varphi (\xi )d\xi ={\rm{\Phi }}({\xi }_{0})-{\rm{\Phi }}({\xi }_{cr,i}^{^{\prime} })$$

with *ξ*_0_ being size of the largest pore section containing the phase ‘L’ at the given *χ*. Notably, the scheme just applied to derive the scanning behavior may be readily continued for more complex variations of the thermodynamic conditions, such as for deducing the scanning loops, etc.

**Figure 4:**
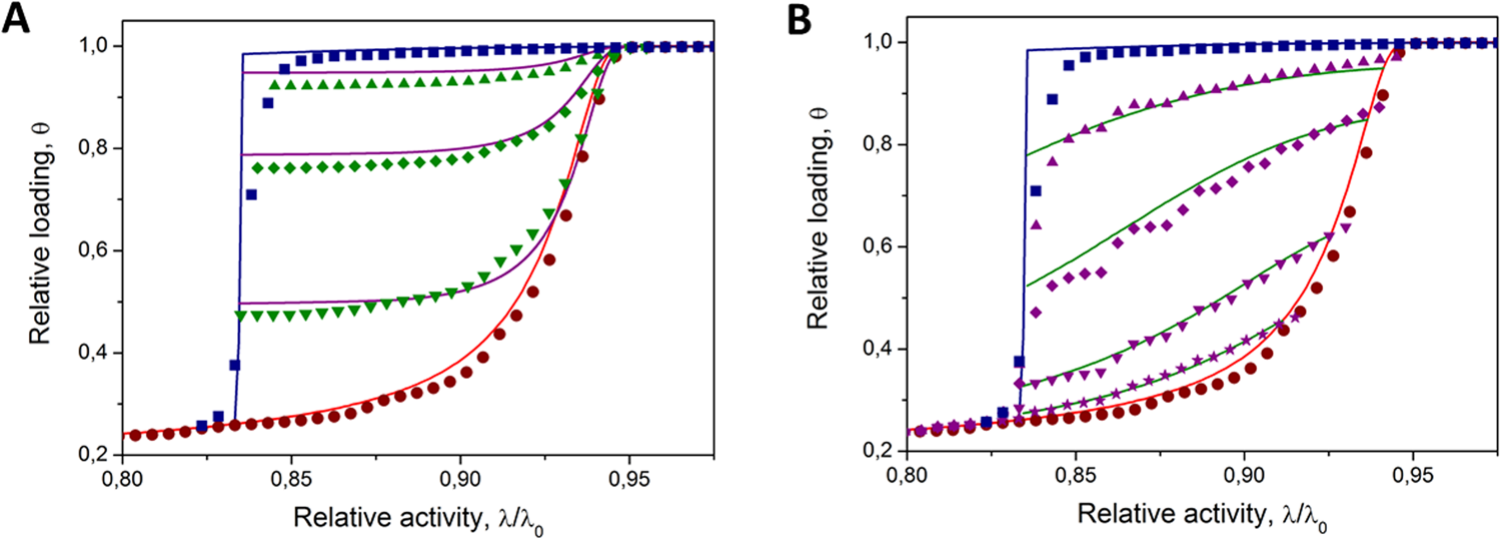
Comparing theory and simulations for scanning behavior in disordered pores. The squares and circles in (**A** and **B**) show the boundary desorption and desorption transitions shown in Fig. 3D. They are complemented in (**A**) by the ascending (adsorption) and in (**B**) by the descending (desorption) scanning data. By the solid lines the scanning curves computed using Eqs 17 and 18 are shown.

In order to check how accurate is the model outlined above, we have also obtained the scanning transitions in the simulations. As an example, Fig. 4 shows the simulation data resulting from the GCMC simulations of a lattice-gas for the system already discussed in the relation to Fig. 3. In addition to the simulation results, the figure shows also the predictions of equations 17 and 18. It turns out that SPM in addition to the boundary transitions nicely reproduces also the scanning behavior. Some minor deviations noted between theory and simulations may have two underlying sources. First of all, these deviations are a penalty for the approximations made upon the derivations, which, however, allowed to keep the final equations as simple as possible. On the other hand, they may result as a consequence of the fact that the kernels used were obtained using ideal pores. It is an open question in which extent thermodynamic fluctuations affect the transitions occurring in along-standing ideal pore sections and in the same sections having neighbours.

### Generic behavior for phase transitions in disordered mesopores

**Figure 5:**
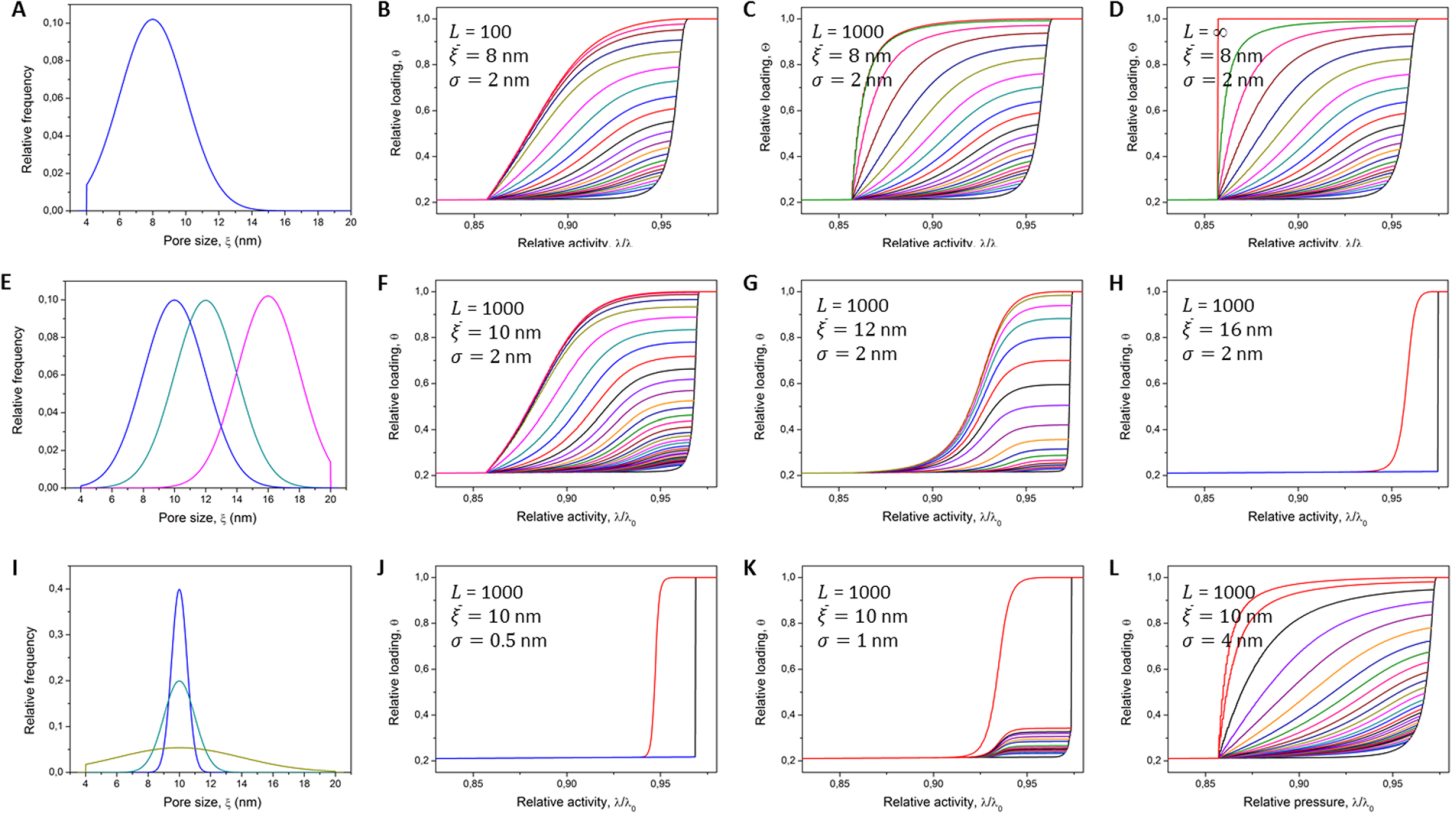
Theoretical predictions for transitions in disordered pores. The figures (**B**–**D**) show the sorption isotherms and desorption scanning curves obtained using SPM for statistically disordered channels with the normal PSD shown in (**A**) ($\overline{\xi }=8$ nm, *σ* = 2 nm) and with the length *L* of 10^2^, 10^3^ and *L* → ∞, respectively. The data of (**F**–**H**) were obtained with the normal PSDs shown in (**E**) with *σ* = 2 nm and with $\overline{\xi }=10$ nm, $\overline{\xi }=12$ nm, and $\overline{\xi }=16$ nm, respectively, and with *L* = 10^3^. The data of (**J**–**L**) were obtained with the normal PSDs shown in (**I**) with $\overline{\xi }/\sigma =\mathrm{1/7}$, *L* = ∞ and with $\overline{\xi }=8$ nm, $\overline{\xi }=9$ nm, and $\overline{\xi }=10$ nm, respectively. In all cases the kernels shown in Fig. 2 were used for computing the isotherms.

The remarkable agreement between the theory outlined and the simulation results obtained proves that this theoretical framework, coupled with the kernels obtained independently, robustly describes the phase transitions in disordered pore systems. Let us now use this framework to discuss the correlations between the phase transitions and geometric characteristics of the porous materials. In particular, we would like to highlight how the transition pathways would change upon varying the total channel length *L*, the average pore size $\overline{\xi }$, and the distribution width *σ*. In Fig. 5 we show a selection of the results showing the boundary hysteresis loops together with the descending scanning curves (as the most interesting ones, the corresponding ascending scans can be found in Supplementary Fig. 4). Note that, in order to not shield an informative part of the desorption transitions at low pressures due to the occurrence of cavitation, we have intentionally introduced a lower cut-off for the pore sizes at 4 nm. In this way the cavitation pressures become lower than the pressure corresponding to the lower closure point of the hysteresis loop. Although the results shown refer to capillary condensation and evaporation phenomena, the qualitative behavior is generic for any other transitions.

The first series of the results (Fig. 5B–D) reveals that, with increasing channel length, the shape of the boundary hysteresis loop varies from type H2(a) for long channels to H2(b) for shorter ones. Here we use the common nomenclature for the sorption isotherms recommended by IUPAC^52^. The adsorption branch remains marginally affected by *L*, while the major impact is on the desorption branch, which exhibits a changeover from a very steep transition for large *L* to a more gradual one for short *L*. Under the condition of ineffective cavitation considered here, the desorption transition is described by equation 13. When the relative gas activity approaches that of the lower closure point of the hysteresis loop, the first non-vanishing term of *θ* is found to be

20 $${\theta }_{d}(\chi )\approx (L\mathrm{/4)}{\rm{\Phi }}({\xi }_{cr,g})$$

Hence, the steepness of the desorption boundary transition (in the same way of the mercury intrusion or of the freezing transition) will be directly proportional to *L*. On the other hand, close to the upper closure point

21 $${\theta }_{d}(\chi )\approx \frac{2}{L}(1-{\rm{\Phi }}({\xi }_{cr,g}))$$

This reveals a more plateau-like behavior for the initial part of the desorption transition for large *L*. Both these features, namely the opposite tendencies close to the lower and upper closure points of the hysteresis loop are nicely seen in Fig. 5B–D.

The family of the scanning curves also nicely reproduces the behavior commonly obtained in the experiments with random porous materials^15, 16, 19, 21, 22^. In contrast to the independent pore model predicting a family of nearly parallel, reversible scanning curves meeting the boundary transitions at different points, the main feature of SPM is that all adsorption and desorption scans meet at the upper and lower closure points of the hysteresis, respectively. Another interesting observation is that the initial slopes of the desorption scans close to the adsorption boundary branch become more gradual with increasing the amount adsorbed. Once again, because in this range of the gas pressures cavitation is ineffective, this finding is readily explained by referring to equation 21. In this equation, however, *L* needs to be replaced by Λ_0_, i.e. by the average length of the domains with the capillary-condensed phase found at the beginning of the the desorption scans. The initial slope of the desorption scans is determined by two quantities, namely by ∂Φ(*ξ*_*cr*,*g*_)/∂*λ* and by Λ_0_. It can be seen that, with increasing gas activity along the boundary line, both quantities change in a way to make the scanning curves more gradual. The strongest effect comes from Λ_0_, which very quickly increases with increasing gas activity due to the merging of the different domains. The shape of the desorption scans with increasing Λ_0_ follows the same pattern as it was obtained previously for the boundary desorption transition upon increasing *L*.

**Figure 6:**
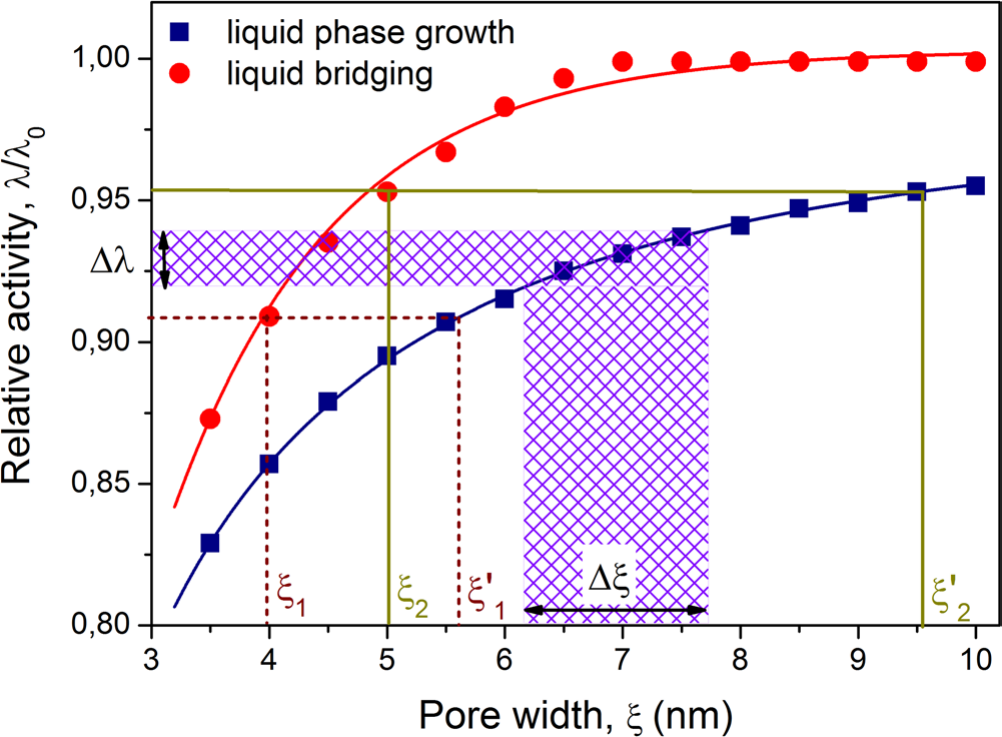
Interplay between nucleation and domain growth. The kernels of Fig. 2C describing nucleation of liquid bridges and domain growth. The broken and solid lines demonstrate that nucleation of a liquid bridge in one section with a pore size *ξ* at a given relative gas activity *λ*/*λ*_0_ triggers liquid condensation in the adjacent sections with the pore size up to *ξ*′. A particular curvature of the kernels leads to an increase of the ratio *ξ*′/*ξ* with increasing *λ*. The shaded bars demonstrate that increasing of the average pore size by keeping the PSD width Δ*ξ* constant leads to transition occurring in a narrower range of Δ*λ*.

Figure 5C,F–H show how the behavior is different if the average pore size $\overline{\xi }$ is increased by keeping the absolute distribution width *σ* unchanged. It turns out that now both boundary transitions become affected by the variation of $\overline{\xi }$. In particular, the adsorption branch becomes steeper and the desorption branch becomes more gradual upon increasing $\overline{\xi }$. The former observation is easily rationalized by noting that shifting PSD towards larger pore sizes results in the onset of nucleation occurring at higher pressures. As demonstrated by Fig. 6 this correspondingly leads to the fact that now larger portions of the pores can simultaneously be filled with the capillary-condensed phase by the axial domain growth. Hence, the transition becomes steeper. The boundary desorption transition is altered in a similar way. As shown in Fig. 6, with increasing $\overline{\xi }$ the corresponding interval Δ*λ* of the gas activities determining the transition range for the given pore size interval decreases. This causes the desorption transition to occur at higher gas activities and in a narrower range Δ*λ*.

It is interesting to note that the initial slope of the descending scans becomes flatter with increasing $\overline{\xi }$. As revealed by Fig. 6, the increase of $\overline{\xi }$ by keeping *σ* constant leads to a strong increase of Λ_0_. As discussed earlier, this gives rise to a stronger pore blocking effect. Eventually, as shown for $\overline{\xi }=16$ nm in Fig. 5H, the adsorption transition becomes so steep that no desorption scans can be obtained anymore. In this regime, condensation in all pores occurs in one step following the formation of a very first liquid bridge. This phenomenon may be observed irrespective of $\overline{\xi }$. Crucial requirement is that PSD should be relatively narrow, see as an example Fig. 5L. This figure reveals also that, for narrow PSDs and for large *L*, one obtains H1 hysteresis loop with two sharp transitions (see, e.g., ref. 28) occurring at the gas pressures corresponding to the equilibrium (gas invasion) and metastable (liquid bridging) transitions in the smallest pores present in the system.

Increasing $\overline{\xi }$ by simultaneously keeping the relative disorder $\sigma /\overline{\xi }$ constant does not preserves the shape of the boundary and scanning isotherms. Their evolution depends on which of the two quantities, Λ_0_ or *σ*, grow quicker with increasing $\overline{\xi }$. In the particular case presented in Fig. 5J–L, the former one dominates and hence stronger pore blocking is observed for larger $\overline{\xi }$.

## Discussion

The results obtained for a linear chain of pores with varying pore diameter along the channel axis are found to be in a remarkable qualitative agreement with the majority of experimental findings obtained for phase equilibria in materials with disordered pore structures. The model naturally leads to the complex cooperativity effects during the transitions by assuming the possibility of a simultaneous action of two transformation mechanisms, nucleation and phase growth. On a microscopic level, they are accounted for by the corresponding kernels, which can independently be obtained with a high accuracy. The general framework furnished with the appropriate kernels may be applied for a variety of systems and under very different thermodynamic conditions. Importantly, the resulting equations describing the transition pathways upon variation of the chemical potential are simple analytical expressions. Hence, they can easily be implemented into the pore structure determination algorithms. Essentially, the governing equations may be interpreted as the application of the BJH scheme, typically used in gas sorption, to the statistical arrays of serially connected individual pores. So far, BJH coupled with the independent pore model remained a pillar for structural characterization of porous solids. The results presented here provide now a closely related alternative allowing for a more reliable approach for structure determination of disordered porous materials. As a robust criterium for selecting between the two approaches one may consider the shapes of the scanning curves. As soon as one finds the scans crossing the boundary curves (the situations when the cavitation pressures are attained on desorption need to be considered separately), independent pore model can be applied, while the scans merging at the hysteresis closure points are the indications towards using the serial pore model.

The capability of the model to describe properly the scanning behavior opens further perspectives for both developing of the in-depth characterization algorithms, for addressing phenomena not yet fully covered, such as unveiling of the fluctuation and network effects, and for correlating the experimental data obtained for different phase transitions. Because one and the same general framework, but decorated with the appropriate kernels describing the phase behaviors on a single-pore level, is applicable for different transitions and for different thermodynamic conditions, the results obtained in different experiments can now be directly compared. Indeed, the equations derived provide a means for the mapping between various experimental data, which essentially turns out to be mapping between different kernels. The potentials of such correlations have frequently been discussed in the literature, but their practical realisation has remained unclear^53–55^.

Another important aspect of the theory presented is the option provided to model more accurately complex physical processes accompanying phase equilibria. Many theoretical approaches aimed at describing physical properties of composite systems, including different phases coexisting in the pore spaces of mesoporous solids, are based on the effective-medium approximations^56^. However, a number of experimentally observed measures or processes can crucially depend not only on the average phase composition, but also on the finer details of the mutual spatial distribution of the coexisting phases. As to the most prominent examples, one may refer to the light or ultrasonic scattering and to diffusion studies in mesoporous materials at different stages of adsorption and desorption or freezing and melting^57–62^. In these experiments it has been unequivocally demonstrated that the light and sound transmissions and the diffusivities measured at one and the same loading, but attained during adsorption and desorption, can be different. In particular, light scattering is controlled by the inhomogeneity of the liquid distribution along the pore space and their number densities^58, 62^. In disordered pore systems, geometric disorder controls the distribution of the effective radii of the scatterers, namely of the domains with the liquid phase. In a close analogy, the diffusivities are the function of how liquid domains are distributed spatially and how they are interconnected via the wetting films^59, 63, 64^. Importantly, the number densities of the different domains (*N*_0_ and ${N}_{0}^{^{\prime} }$) as well as their spatial extensions (Λ_0_ and ${{\rm{\Lambda }}}_{0}^{^{\prime} }$), which are different for different preparation histories of one and the same average phase composition, are readily obtained within the model outlined here. Hence, the model provides an effective approach for predicting scattering and transport properties along different transition pathways.

With all potentials highlighted, there are still several open questions which need to be addressed in future studies. In the first line, it is critical to establish how accurately can the single pore model describe real porous solids with the pore spaces of higher dimensions, rather than one-dimensional pore systems considered here. In this respect, no distinctive conclusions may be inferred by referring to percolation theories because the nucleation processes may have substantial impact on the phase equilibria. At the same time, some features inherent in percolation problems are of relevance, in particular in determining the phase growth processes. The most essential difference between the serial and independent pore models is the fact that the former one takes account of the cooperativity effects in phase transitions. Most notably, they control growth of the phase domains at given thermodynamic conditions. Hence, for discussing the dimensionality effects upon phase equilibria it is absolutely essential to consider in all details what happens at the junctions between different pores. On the one hand, the junctions may provide an effective mechanism for transferring the phase state to the neighbouring pores. For example, a junction with a relatively large size can promote gas invasion. In this case, this will render the overall behavior even further from that predicted by the independent pore model. On the other hand, the junctions may hinder phase growth, like the same junction of a large size can prohibit advanced adsorption. Hence, the overall behavior may become to be shifted towards the independent pore model. With the two examples mentioned, it is evident that one and the same junction may have opposite impacts upon different transition mechanisms. While one of the transition branches may develop more features inherent in the independent pore model, the other one may become affected in the opposite way. Hence, in order to gain deeper insight into the dimensionality effects, more close attention needs to be paid first to assess geometric features of the pore junctions and to understand their role in promoting or hindering the transfer of phase state to adjacent pores. These studies may also shed light on the question whether the network effects can be reliably determined from the experimental data. Another interesting problems which have become apparent during this work are how accurate the conventionally obtained kernels are for describing phase transitions in pores with complex pore geometries and how to relate the length parameter *L* of the present work to real extensions of porous materials.

## Methods

### GCMC simulations of gas sorption

A simple cubic single occupancy lattice-gas model in an external field and with nearest neighbor interactions was considered. The external field serves to model the pore geometry confining the lattice gas. The Hamiltonian for this model is given by

22 $$ {\mathcal H} =-\epsilon \sum _{\langle i,j\rangle }{n}_{i}{n}_{j}+\sum _{i}{n}_{i}{\varphi }_{i},$$

where *ε* denotes the nearest neighbor interaction strength, 〈*i*, *j*〉 indicates the sum over all nearest neighbor bonds, *n*_*i*_ ∈ {0, 1} is the occupation number, and *ϕ*_*i*_ the external field at the lattice node *i*. For lattice nodes adjacent to pore walls, the external field is chosen to be *ϕ*_*i*_ = 3*ε*. This value is based roughly on the energy minimum experienced by a Lennard-Jones-(12, 6) atom close to a solid constituted by the same atoms^31^. For all other lattice nodes *ϕ*_*i*_ equals zero. The lattice constant was chosen to be 0.5 nm. The Grand canonical Monte Carlo (GCMC) simulations were performed using the Metropolis algorithm^65^, which, in order to change the state of the lattice gas towards the equilibrium state, at each step attempts to reverse the occupancy of every node with the probabilities

23 $${p}_{{\rm{add}}/{\rm{remove}}}=\,{\rm{\min }}[\mathrm{1,}\,{e}^{\mp \beta ({\rm{\Delta }}E-\mu )}],$$

where *β* = 1/(*kT*), Δ*E* is the energy change between the new and the old system state and *μ* denotes the chemical potential. In order to obtain the sorption isotherms, the relative activity $\lambda /{\lambda }_{0}={e}^{\beta (\mu -{\mu }_{0})}$ is used as thermodynamic quantity of variation, where the subscript zero indicates bulk saturation. For the lattice gas model the chemical potential at saturation is known analytically as *μ*_0_ = −*Zϵ*/2, *Z* = 6 being the coordination number of the lattice. Apart from the effect of gas imperfection, the relative activity is equivalent to the relative pressure^31^. Starting from a low relative activity with an initial system totally empty, then gradually increasing the activity under quasi-equilibrium conditions using the last system state of the previous activity as initial guess for the next one, with the help of the Metropolis algorithm yielding the most probable system states, the average loading can be obtained as a time-average of the occupation numbers. The Monte Carlo simulations were performed at a relative temperature *β*/*β*_0_ = 2/3, where *β*_0_ ≈ 9*ϵ*/8 denotes the bulk critical temperature^66^. With this, the interaction strength can be determined as *βϵ* = 4/3.

The kernels for liquid bridging were obtained by finding the relative transition activity for capillary condensation in ideal open-ended slit pores with a length of 10 nanometers. The kernels for advanced adsorption and desorption were achieved by means of the capillary condensation and evaporation transition in ideal channels of the same length, but capped at one end. Finally, the cavitation kernels were found corresponding to the capillary evaporation transition in 10 nanometer long ideal slit pores closed at both ends. To study the sorption isotherms in disordered pores, the simulation results were averaged over 20 sample pores composed of 1000 conjoined sections of different pore diameter randomly drawn from PSD and a length of 10 nanometers.

### MC simulations of freezing and melting

The freezing and melting transitions were addressed using an approach described in ref. 67. It employs a simple cubic lattice model in an external field with nearest-neighbor interactions only^46^. Without the external field included, whose purpose is to model the presence of the pore walls, it resembles the Kossel-Stranski model widely used to study bulk crystal growth processes^68, 69^. The configurational energy is given by

24 $$H=\sum _{i}(-{N}_{ii}{\varphi }_{ii}-{N}_{is}{\varphi }_{is}+{N}_{i}{F}_{i})+\sum _{i\ne k}(-\frac{1}{2}{N}_{ik}{\varphi }_{ik}),$$

where the subscripts *i* and *k* can assume either of two values, namely *c* if the site considered is in the crystal state and *f* if it is in the fluid state, the subscript *s* stands for solid (pore walls), *N*_*ik*_ is the number of the *ik* bonds, *N*_*i*_ is the number of sites of type *i*, and −*ϕ*_*ik*_ is the potential energy of the nearest neighbor interaction between *i*-th and *k*-th sites, *F*_*c*_ and *F*_*f*_ are the internal energies of the crystal and fluid sites, respectively. With *H* given by equation 24, the probabilities to convert a randomly selected site form the fluid to crystal (*p* ^+^) or from the crystal to fluid (*p* ^−^) states resulted as

25 $${p}^{\pm }=\frac{1}{2}exp\{\mp \frac{1}{2}\frac{{\rm{\Delta }}G}{kT}\}$$

with Δ*G* defined as

26 $${\rm{\Delta }}G=-L(\frac{{n}_{ii}}{3}-\frac{T}{{T}_{0}})+{{\rm{\Omega }}}_{c}{\rm{\Delta }}{n}_{cs}.$$

In equation 26, *L* = 3(*ϕ*_*cc*_ − *ϕ*_*ff*_) is the heat of fusion, *T*_0_ is the bulk equilibrium transition temperature, *n*_*ii*_ is the resulting number of neighbors of the same type after the site conversion (*f* for the crystal to fluid and *c* for the fluid to crystal conversions, respectively), Δ*n*_*cs*_ is the change in the crystal-solid bond numbers upon the conversion, and Ω_*c*_ = [−*ϕ*_*cs*_ + *ϕ*_*fs*_ + (*ϕ*_*cc*_ − *ϕ*_*ff*_)/2]. For the simulations presented in this work, we assumed *ϕ*_*ff*_ = *ϕ*_*cf*_ and Ω_*c*_ = 3*L*. The latter ensured the formation of the liquid layer between the pore walls and the crystal core in the pore interior in line with the experimental evidence. The results were obtained with *L* = 10 kJ/mol and *T*_0_ = 278 K.

In the simulations, the sites representing the pore walls were distributed on a cubic lattice to form channels with desired configurations. Their state did not change during the simulations. The channels openings had direct contact to the crystallizer, whose sites for temperatures below *T*_0_ were always kept in the crystal state. For the sake of convenience the site length was fixed to 0.5 nm, thus all dimensions shown in the figures are in nm. For establishing the transition kernels, the following geometries were used. The ice invasion from the pore openings was studied with the cylindrical pores open at both ends. The temperatures of homogeneous ice nucleation were assessed using long channels closed at both ends. To obtain the temperature for the formation of liquid bridges once again long channels open at both ends in contact with the bulk crystallizer were used. Finally, the equilibrium temperature for the growth of the liquid domains were obtained using capped cylindrical pores. In the latter case, the occurrence of a liquid non-frozen layer at the capped end removed the metastability related with the formation of the liquid bridges. To study the transitions in disordered pores, long channels composed of 300 sections with different pore diameters (see PSD in Fig. 3A) and each with a length of 2.5 nm were used. The results shown in Fig. 3E were averaged over 20 different realizations of the channel geometric configuration.

### Data availability

The data that support the findings of this study are available from the corresponding author upon request.

## Electronic supplementary material

Supplementary Information 
