# Supplementary material for: Phase transitions in disordered mesoporous solids
Source: Sci Rep. 2017 Aug 3;7:7216. doi: 10.1038/s41598-017-07406-2 (PMC5543148; doi:10.1038/s41598-017-07406-2)
Supplement: Supplementary file 1 — Supplementary Information [file 41598_2017_7406_MOESM1_ESM.pdf]

Phase transitions in disordered mesoporous solids

Daniel Schneider, Daria Kondrashova, and Rustem Valiullin*

University of Leipzig, Institute for Experimental Physics II, Leipzig, Germany

E-mail: valiullin@uni-leipzig.de

*To whom correspondence should be addressed

*Supplementary information

Additional remarks to the SPM theory, boundary curves

- **Mean length λ of a domain grown from a seed**

Considering the mean probability of phase growth from one pore segment to an adjacent segment $p_g(\chi)$, the mean length λ of a domain grown from a seed in a channel of length L can be expressed as

$$\lambda(\chi) = 2\sum_{k=1}^{L/2} p_g^k = 2p_g \frac{1 - p_g^{L/2}}{1 - p_g}, \qquad (1)$$

where the growth in both possible directions is taken into account by the prefactor 2 and boundary effects at the pore ends are accounted for approximately by the mean possible maximum grow length in one direction, $L/2$. Fig. 1 shows the evolution of λ upon variation of p_g for different pore lengths.

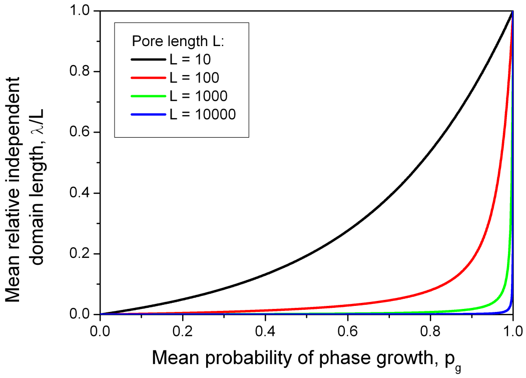

Figure 1: Mean relative independent domain length versus mean probability of phase growth according to Eq. 1.

- **Relative phase composition θ**

By randomly placing a phase domain of length $\lambda(\chi)$ in a pore of length L, the probabil-

ity that a pore segment remains unaffected (not covered) by the domain is $P_1 = 1-\lambda/L$ (in disregard of boundary effects at the pore ends). Next, consider $n(\chi)$ nucleation events occurring randomly in the pore space, each growing to a domain of mean length λ. Then the probability that a pore segment still remains unaffected by these domains is P_1^n and, thus, the complementary probability, in other words, the fraction of pore segments covered by the domains is

$$\theta(\chi) = 1 - \left(1 - \frac{\lambda(\chi)}{L}\right)^{n(\chi)} \tag{2}$$

Considering $L \gg 1$, this equation can be rewritten as

$$\theta(\chi) \xrightarrow{L\gg 1} 1 - e^{-\frac{n\lambda}{L}}. \tag{3}$$

- **Mean number N_0 and length Λ_0 of continuous domains**

Consider placing n domains of length λ randomly in an interval of length L. Then, for $n \gg 1$ and by disregarding the boundary effects, the distance s between the domain centers is distributed according to [see, e.g., T. Huillet, On a deposition process on the circle with disorder. Advances in Applied Probability, 2004, 36, 996-1020]

$$p(s) = \rho e^{-\rho s}, \tag{4}$$

where $\rho = n/L$ is the number density of the seeds. The domains are allowed to overlap. Thus, there is the possibility that two or more domains merge into one larger domain. Taking this into account, the actual mean number of the continuous domains is given by

$$N_0 = n \int_{\lambda}^{\infty} p(s)ds = ne^{-\frac{n\lambda}{L}}. \quad (5)$$

Here we have purposefully integrated over the distances between two domains which are larger than λ (distances, where domains do not overlap, see Fig. 2). Using equation Eq.(3), for $L \gg 1$, the mean number of continuous domains can be written as follows;

$$N_0 = n(1 - \theta). \quad (6)$$

Finally, the mean length of the domains taking into account merging and overlap equals the mean total length occupied by all the domains, which is θL, divided by the mean number of domains, N_0:

$$\Lambda_0 = \frac{\theta L}{N_0}. \quad (7)$$

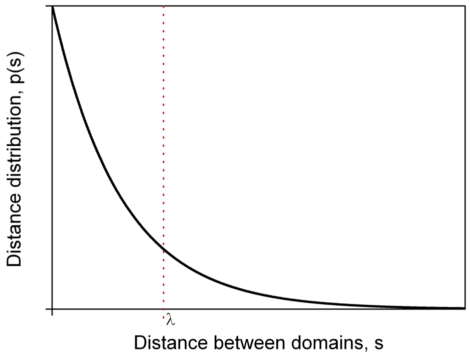

Figure 2: Distribution of distances between the centers of equal-sized domains randomly placed in an interval of length L. All distances greater than the domain length λ indicate that there is no overlap between the two domains.

- **Limit of long pores**

In long and/or heavily disordered pores, the coarse-graining approach used in the SPM theory involves a large number of pore segments L. Being difficult to access in experiments, it is convenient to drop L from the calculations. For $L \gg 1$, the independent domain length (Eq.(1)) can be approximated as follows;

$$\lambda(\chi) \xrightarrow{L \gg 1} \frac{2p_g}{1-p_g}. \tag{8}$$

With boundary effects neglectable for large L, $p_n = nL$, and the mean relative phase composition (Eq.(3)) can be expressed as

$$\theta = 1 - e^{-p_n \lambda}. \tag{9}$$

To achieve a measure of the mean number and mean length of the phase domains, N_0 and Λ_0, a density-like description can be used, yielding;

$$P_0 = p_n(1-\theta) \qquad \Lambda_0 = \frac{\theta}{P_0}. \tag{10}$$

- **Phase composition curves of boundary hysteresis loops**

Recalling that $p_i = \Phi[\xi_{cr,i}]$ and $p'_i = 1 - \Phi[\xi'_{cr,i}]$, where $\xi^{(\prime)}_{cr,i}(\chi) = (\chi^{(\prime)}_{i,tr})^{-1}(\chi)$ denotes the inverse kernel functions, the phase composition curves can be written as function solely of the pore geometry (Φ and L), the thermodynamic conditions (χ and n_b) and the kernels as follows;

– The ascending boundary curves can be expressed as

$$\theta_a(\chi;\Phi,L,n_b,\{\chi_{i,tr}\}) = 1-\left[1-\frac{2p_g}{L}\frac{1-p_g^{L/2}}{1-p_g}\right]^{p_nL+n_b} \tag{11}$$

$$\xrightarrow{L\to\infty} \theta_a(\chi;\Phi,\{\chi_{i,tr}\}) = 1-e^{-\frac{2p_np_g}{1-p_g}}. \tag{12}$$

– The descending boundary curves can be written as

$$\theta_d(\chi;\Phi,L,n'_b,\{\chi'_{i,tr}\}) = \left[1-\frac{2p'_g}{L}\frac{1-(p'_g)^{L/2}}{1-p'_g}\right]^{p'_nL+n'_b}. \tag{13}$$

For $L\to\infty$, in the case of prevalent nucleation, Eq. (13) can be simplified to

$$\theta_d(\chi;\Phi,\{\chi'_{i,tr}\}) = e^{-\frac{2p'_np'_g}{1-p'_g}}, \tag{14}$$

and without nucleation, but solely phase growth ($p'_n = 0$),

$$\theta_d(\chi;\Phi,L,\chi'_{g,tr}) = 1-\frac{2}{L}\frac{p'_g}{1-p'_g}. \tag{15}$$

Step-by-step implementation of the SPM theory, scanning behavior

Ascending scan

Considering a point (χ_0,θ_d) on the descending boundary transition line, the following information about the phase composition in a disordered pore of length L and a PSD of $\phi(\xi)$ can be extracted;

- The mean number of continuous domains of phase 'G' is

$$N'_0 = n'_0 \theta_d, \tag{16}$$

where n'_0 denotes the number of seeds for phase growth either due to nucleation events or boundary conditions.

- The mean length of the phase-'G'-domains can be expressed as

$$\Lambda'_0 = \frac{(1-\theta_d)L}{N'_0}. \tag{17}$$

- The diameter of the smallest pore that contains phase 'G',

$$\xi_0 = (\chi'_{tr,g})^{-1}(\chi_0), \tag{18}$$

where $(\chi'_{tr,g})^{-1}$ is the inverse kernel for growth of phase 'G', for $\chi'_{tr,g}$ is typically larger than $\chi'_{tr,n}$, yielding the smallest possible pore.

In summary, this means that in the channel on average N'_0 phase-'G'-domains of mean length Λ'_0 and covering only pore segments with a diameter larger than ξ_0 exist. This information can now be used to study the ascending scanning behavior upon gradually increasing χ from χ_0 towards a state, where the entire pore is filled with phase 'L'. Thus, during the rise of χ, the gradual transition of the phase-'G'-domains into phase 'L' is observed. Since we are working with mean values, one domain stands exemplary for all the others.

At first the mean transition probabilities ('G' $\rightarrow$ 'L') can be found as

$$p_{\mathrm{as},i}(\chi,\chi_0) = \frac{1}{Z}\int_{\xi_0(\chi_0)}^{\infty} f_i(\chi,\xi)\phi(\xi)d\xi = \frac{1}{Z}\left\{\Phi[\xi_{cr,i}(\chi)] - \Phi[\xi_0(\chi_0)]\right\}, \tag{19}$$

where $f_i(\chi,\xi) = \mathrm{Heaviside}[\chi - \chi_{tr,i}(\xi)]$ is the function describing whether a pore segment with diameter ξ can be filled by phase 'L' via mechanism $i \in \{n, g\}$, $\xi_{cr,i} = \chi^{-1}_{tr,g}(\chi)$ is the

inverse kernel function of mechanism i at χ and $Z = \int_{\xi_0}^{\infty} \phi(\xi) d\xi$. The integral boundaries in Eq. (19) take into account approximately the PSD of the initial phase-'G'-domains, which differs from the PSD of the entire pore. With the adjusted mean transition probabilities in hand, we can follow the procedures leading to the ascending boundary curve with minor changes to obtain the ascending scanning curve.

- Consequently, the mean number of seeds of phase 'L' in a phase-'G'-domain results to

$$n(\chi, \chi_0) = p_{as,n}\Lambda_0' + n_b, \tag{20}$$

where $n_b = 1 - p_{as,n}$, considering that all of the domains (except at the pore openings) are surrounded by the complementary phase.

- The mean length a domain grows from a phase-'L'-seed can be expressed as

$$\lambda(\chi, \chi_0) = 2p_{as,g}\frac{1 - p_{as,g}^{\Lambda_0'/2}}{1 - p_{as,g}}. \tag{21}$$

- Next, the phase composition of the domain initially covered by phase 'G' changes with increasing χ according to

$$\theta(\chi, \chi_0) = 1 - \left(1 - \frac{\lambda}{\Lambda_0'}\right)^n. \tag{22}$$

Finally, the ascending scanning curve is obtained as the following union;

$$\theta_{as}(\chi, \chi_0) = \theta_d(\chi) + \theta(\chi, \chi_0) - \theta_d(\chi)\theta(\chi, \chi_0). \tag{23}$$

Descending scan

Considering a point (χ_0, θ_a) on the ascending boundary transition line, the following information about the phase composition in a disordered pore of length L and a PSD of $\phi(\xi)$ can

be extracted.

- The mean number of continuous domains of phase 'L' is

$$N_0 = n_0(1 - \theta_a), \tag{24}$$

where n_0 denotes the number of seeds for phase growth either due to nucleation events or boundary conditions.

- The mean length of the phase-'L'-domains can be expressed as

$$\Lambda_0 = \frac{\theta_a L}{N_0}. \tag{25}$$

- The diameter of the largest pore that contains phase 'L',

$$\xi_0 = (\chi_{tr,g})^{-1}(\chi_0), \tag{26}$$

where $(\chi_{tr,g})^{-1}$ is the inverse kernel for growth of phase 'L', for $\chi_{tr,g}$ is typically smaller than $\chi_{tr,n}$, yielding the largest possible pore.

In summary, this means that in the channel on average N_0 phase-'L'-domains of mean length Λ_0 and covering only pore segments with a diameter smaller than ξ_0 exist. This information can now be used to study the descending scanning behavior upon gradually decreasing χ from χ_0 towards a state, where the entire pore is filled with phase 'G'. Thus, during the decline of χ, the gradual transition of the phase-'L'-domains into phase 'G' is observed. Since we are working with mean values, one domain stands exemplary for all the others.

At first the mean transition probabilities ('L' $\rightarrow$ 'G') can be found as

$$p'_{\mathrm{ds},i}(\chi, \chi_0) = \frac{1}{Z}\int_0^{\xi_0(\chi_0)} f_i(\chi, \xi)\phi(\xi)d\xi = \frac{1}{Z}\left\{\Phi[\xi_0(\chi_0)] - \Phi[\xi'_{cr,i}(\chi)]\right\}, \tag{27}$$

where $f_i(\chi,\xi) = \text{Heaviside}[\chi - \chi'_{tr,i}(\xi)]$ is the function describing whether a pore segment with diameter ξ can be filled by phase 'G' via mechanism $i \in \{n, g\}$, $\xi'_{cr,i} = (\chi'_{tr,g})^{-1}(\chi)$ is the inverse kernel function of mechanism i at χ and $Z = \int_0^{\xi_0} \phi(\xi) d\xi$. The integral boundaries in Eq. (27) take into account approximately the PSD of the initial phase-'L'-domains, which differs from the PSD of the entire pore. With the adjusted mean transition probabilities in hand, we can follow the procedures leading to the descending boundary curve with minor changes to obtain the descending scanning curve.

- Consequently, the mean number of seeds of phase 'G' in a phase-'L'-domain results to

$$n'(\chi, \chi_0) = p'_{ds,n}\Lambda_0 + n'_b, \tag{28}$$

  where $n_b = 1 - p'_{ds,n}$, considering that all of the domains are surrounded by the complementary phase.

- The mean length a domain grows from a phase-'G'-seed can be expressed as

$$\lambda'(\chi, \chi_0) = 2p'_{ds,g} \frac{1 - (p'_{ds,g})^{\Lambda_0/2}}{1 - p'_{ds,g}}. \tag{29}$$

- Next, the phase composition of the domain initially covered by phase 'L' changes with decreasing χ according to

$$\theta(\chi, \chi_0) = \left(1 - \frac{\lambda'}{\Lambda_0}\right)^{n'}. \tag{30}$$

Finally, the descending scanning curve is obtained as the following intersection;

$$\theta_{ds}(\chi, \chi_0) = \theta_a(\chi)\theta(\chi, \chi_0). \tag{31}$$

Wetting films

In the case of a wetting transition at the pore walls, the relative phase composition has to be adjusted for the share of the wetting layers as follows;

$$\theta_{\text{tot}} = \theta + (1 - \theta)\theta_w, \tag{32}$$

where θ_w indicates the mean volume fraction of the wetting layers in a pore segment. Regarding the GCMC simulations in disordered pores, θ_w was obtained with the best fit of

$$\theta_w(\chi) = \frac{4a}{\sqrt{b - ln(\chi)}} \left(1 - \frac{a}{\sqrt{b - ln(\chi)}}\right) \tag{33}$$

in the regime where the layers are already established, but according to the kernels, before capillary transitions occur to a significant amount (see Fig. 3). The fitting function was obtained by substituting the Halsey equation $\delta(\chi) = c/\sqrt{b - ln(\chi)}$ for the wetting layer thickness δ[?] into

$$\theta_w = \frac{V - V_{gas}}{V} = \frac{d^2 - (d - 2\delta)^2}{d^2} = 4\frac{\delta}{d}\left(1 - \frac{\delta}{d}\right), \tag{34}$$

assuming pore segments of cylindrical shape.

Note that the wetting films also affect the phase nucleation and growth processes. These effects, however, are already incorporated and thus, accounted for, in the respective kernels.

Adsorption scanning curves

Fig. 4 shows the theoretical predictions for transitions in various disordered pores including the adsorption scanning curves.

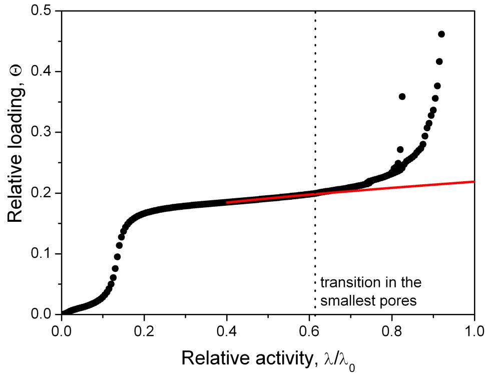

Figure 3: Gas sorption isotherms (black dots) in a disordered pore showing a wetting transition and the corresponding best fit of Eq. (33) (red line).

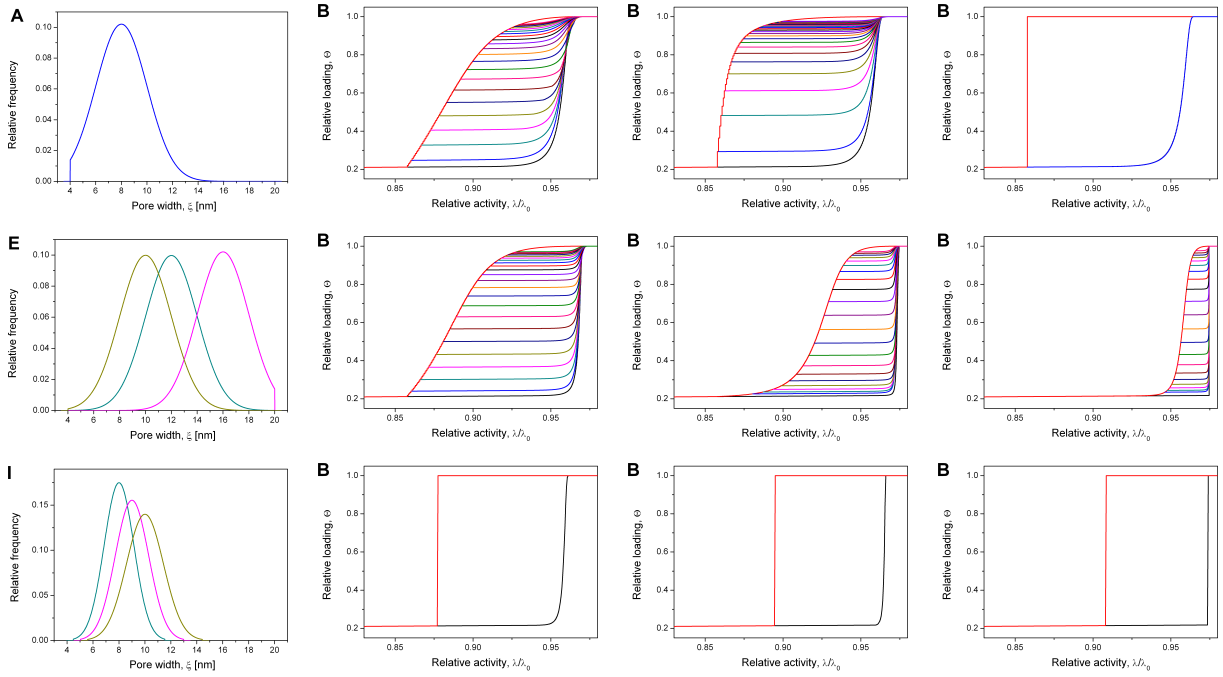

Figure 4: Theoretical predictions for transitions in disordered pores. Supporting figure to Fig. 4 replacing the desorption scanning with adsorption scanning curves. For more information see Fig. 4.
